# Wear Behavior of an Unstable Knee: Stabilization via Implant Design?

**DOI:** 10.1155/2014/821475

**Published:** 2014-09-09

**Authors:** Jörn Reinders, Robert Sonntag, Jan Philippe Kretzer

**Affiliations:** Laboratory of Biomechanics and Implant Research, Clinic for Orthopedics and Trauma Surgery, Center for Orthopedics, Trauma Surgery and Spinal Cord Injury, Heidelberg University Hospital, Schlierbacher Landstraße 200a, 69118 Heidelberg, Germany

## Abstract

*Background*. Wear-related failures and instabilities are frequent failure mechanisms of total knee replacements. High-conforming designs may provide additional stability for the joint. This study analyzes the effects of a ligamentous insufficiency on the stability and the wear behavior of a high-conforming knee design. *Methods*. Two simulator wear tests were performed on a high-conforming total knee replacement design. In the first, a ligamentous-stable knee replacement with a sacrificed anterior cruciate ligament was simulated. In the second, a ligamentous-unstable knee with additionally insufficient posterior cruciate ligament and medial collateral ligament was simulated. Wear was determined gravimetrically and wear particles were analyzed. Implant kinematics was recorded during simulation. *Results*. Significantly higher wear rates (*P* ≤ 0.001) were observed for the unstable knee (14.58 ± 0.56 mg/10^6^ cycles) compared to the stable knee (7.97 ± 0.87 mg/10^6^ cycles). A higher number of wear particles with only small differences in wear particle characteristics were observed. Under unstable knee conditions, kinematics increased significantly for translations and rotations (*P* ≤ 0.01). This increase was mainly attributed to higher tibial posterior translation and internal rotations. *Conclusion*. Higher kinematics under unstable test conditions is a result of insufficient stabilization via implant design. Due to the higher kinematics, increased wear was observed in this study.

## 1. Introduction

Implant failure due to massive polyethylene (PE) wear and wear-associated aseptic loosening has been one of the main challenges concerning total knee replacements (TKRs) in the past decades [1–3]. This has led to extensive research aimed at increasing the wear performance of TKR. Experimental wear studies showed that improvements in manufacturing, sterilization, and design optimization can be used to increase the wear resistance of TKR [4–7]. Therefore, clinical implementation of these technical improvements should increase the longevity of currently used TKR.

Clinically, failure analysis of currently available TKR confirms a reduction in wear-related revisions [8–10]. Nevertheless, wear remains a critical issue especially for the long-term success of TKR. As wear-related revisions decrease, other failure mechanisms become more relevant. Instabilities have become one of the most frequent failure mechanisms in TKR [8, 11] as they are often seen in the short and midterm (<5 years).

Aetiology of instabilities is often multifactorial, but a relevant portion can be attributed to ligamentous insufficiency [12]. A clinical solution that may address ligamentous instabilities is the use of a high-conforming knee design. However, concerns exist related to the higher grade of coupling. Increased bone-implant loading may be assumed. Additionally, conformity influences contact patterns and consequently kinematics as well as wear of TKR [13–15]. Until now, experimental wear studies cannot clearly answer whether high conformity has beneficial [16] or adverse [17, 18] effects on wear. This is related to the superimposing effects of surface stress, wear area, and resulting kinematics on the wear behavior.

Wear testing should be carried out based on the clinical background of expected loading. Patient collectives designated for the use of a high-conforming knee design differ from patient collectives designated for the use of an unconstrained knee design. The use of a high-conforming knee design may be plausible if a ligamentous insufficiency already exists or is anticipated during one's lifetime. This should be considered during wear testing.

The aim of this study is to analyze the effects of a ligamentous insufficiency on the stability and the wear behavior of a high-conforming knee design.

## 2. Material and Methods

### 2.1. Simulation

**Figure 1:**
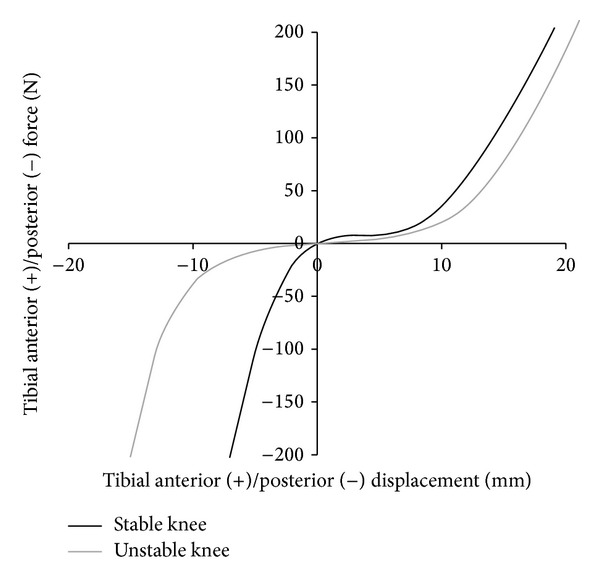
Anterior/posterior restraint in this study based on cadaveric studies [20–22]. The plot shows the level of restraint related to the characteristics of the passive structures (soft tissues, capsule, and ligaments).

**Figure 2:**
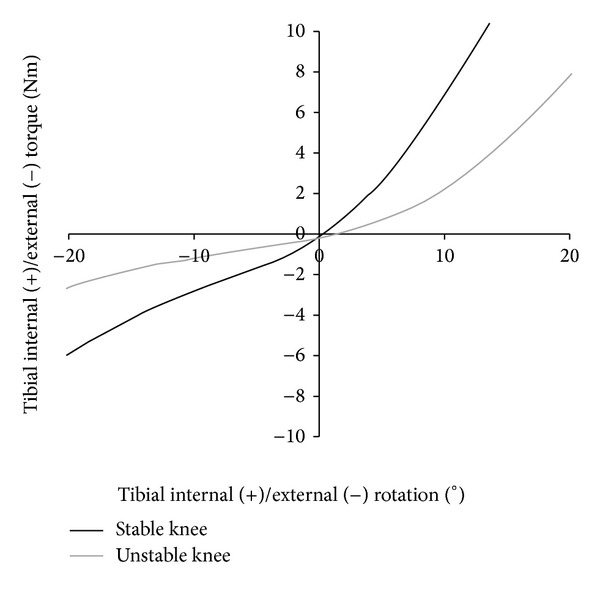
Internal/external restraint in this study based on cadaveric studies [20–22]. The plot shows the level of restraint related to the characteristics of the passive structures (soft tissues, capsule, and ligaments).

Two knee wear tests were performed on an AMTI knee simulator (Model KS2-6-1000, Advanced Mechanical Technology Inc., Watertown, MA, USA) using two different restraint characteristics. Restraint characteristics are defined by the restraint of the passive structures (ligaments, soft tissue, and capsule) which are based on* in vitro* laxity measurements [19–21]. In the first scenario, a stable TKR was defined with an absent anterior cruciate ligament and otherwise intact ligament structures [22]. In the second scenario, a ligamentously insufficient stabilized TKR (unstable TKR) was defined with an absent anterior cruciate ligament (ACL), insufficient posterior cruciate ligament (PCL), and medial collateral ligament (MCL). Simulated ligament characteristics are shown in Figures 1 and 2.

Disregarding restraint characteristics, wear tests were run with force-controlled parameters according to ISO 14243-1:2009 with an extension/flexion of 0°–58°, a maximum axial load of 2600 N, anterior/posterior forces of −265 to 110 N, and internal/external torques of −1 to 6 Nm. Axial forces were transmitted with a 7% medial offset of the tibial plateau width in order to achieve physiologically higher forces on the medial plateau.

### 2.2. Materials

For wear testing, a deep-dished, ultracongruent (manufacturer specification), cruciate-substituting implant design (TC-Plus, Smith  &  Nephew, Baar, Switzerland) was used. PE-components were irradiated in an inert gas atmosphere (25–37 kGy). The inserts were presoaked in bovine serum prior to the simulation. Inserts were gravimetrically measured on a weekly basis until the incremental increase in weight was less than 10% of the total cumulative weight increase. In detail, components were presoaked for 105 days (stable conditions) and for 132 days (unstable conditions). Every wear test consisted of three specimens plus one axially loaded soak control. Tests were run for a total of 5 million cycles in diluted bovine serum (PAA Laboratories GmbH, Pasching, Austria) with a protein content of 20 g/L. The testing fluid (250 mL) was tempered to 37°C during the simulation. As additives, sodium azide (1.85 g/L) and ethylenediamine tetra-acetic acid (7.44 g/L) were used to prevent bacterial growth and to minimize calcium phosphate layers, respectively.

### 2.3. Wear Analysis

At intervals of 500,000 cycles, the wear testing was interrupted to replace the bovine serum and determine the PE wear mass. Components were cleaned and measured gravimetrically according to ISO 14243:2:2009. At the end of each test, wear particles were analyzed using acid digestion according to previously published methods [23, 24]. Particles were analyzed on filters with a pore size of 20 nm using high resolution SEM (FEGSEM, Leo 1530, Leo, Oberkochen, Germany) at a magnification of 25,000. Size, morphology, and number of particles were determined [23–26] using an image analyzing software (Leica QWin V3, Leica Microsystems, Wetzlar, Germany). Implant kinematics (anterior/posterior translations and internal/external rotations) was recorded during the simulation and analyzed in each interval (every 500,000 cycles) using the simulator's own measurement system. Wear areas were documented photographically.

### 2.4. Statistics

Wear rates and kinematics were compared using Student's *t* test with a level of significance set at *P* < 0.05. All data is shown with mean ± standard deviation.

Wear particle characteristics are based on a high number of wear particles. Effect size was calculated according to Cohen [27] in order to compare wear particle characteristics between both tests.

## 3. Results

**Figure 3:**
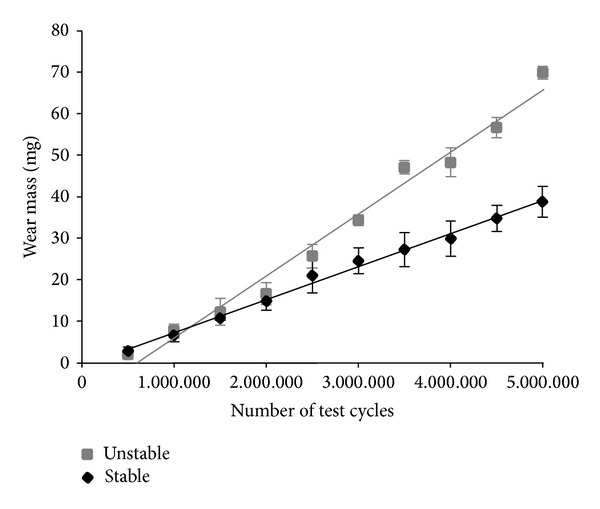
Wear progression for ligamentous-stable and ligamentous-unstable test conditions.

Wear progression of both tests is shown in Figure 3. Simulation under stable knee conditions resulted in a wear rate of 7.97 ± 0.87 mg/10^6^ cycles. Simulation of an unstable knee resulted in a significantly increased wear rate of 14.58 ± 0.56 mg/10^6^ cycles (*P* ≤ 0.001).

**Figure 4:**
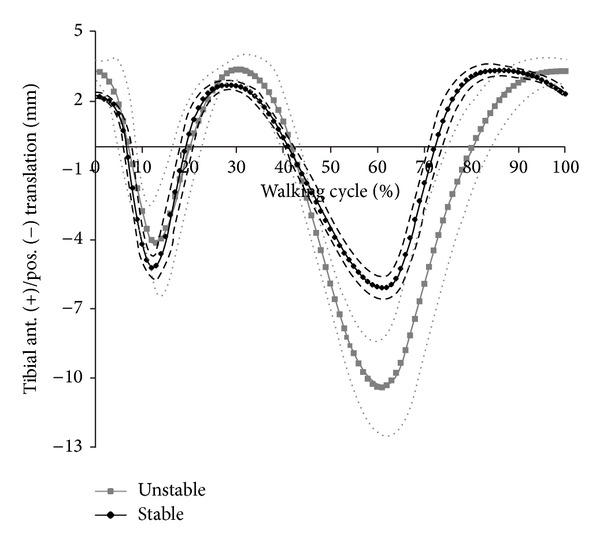
Tibial anterior and posterior translation for ligamentous-stable and ligamentous-unstable test conditions (dashed line = standard deviation).

**Figure 5:**
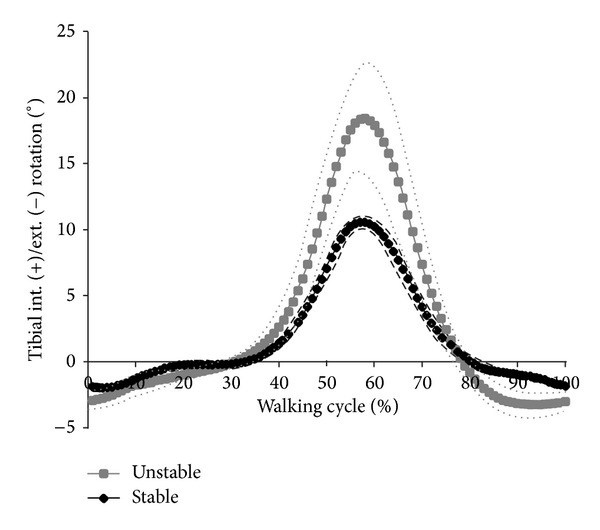
Tibial internal and external translation for ligamentous-stable and ligamentous-unstable test conditions (dashed line = standard deviation).

**Figure 6:**
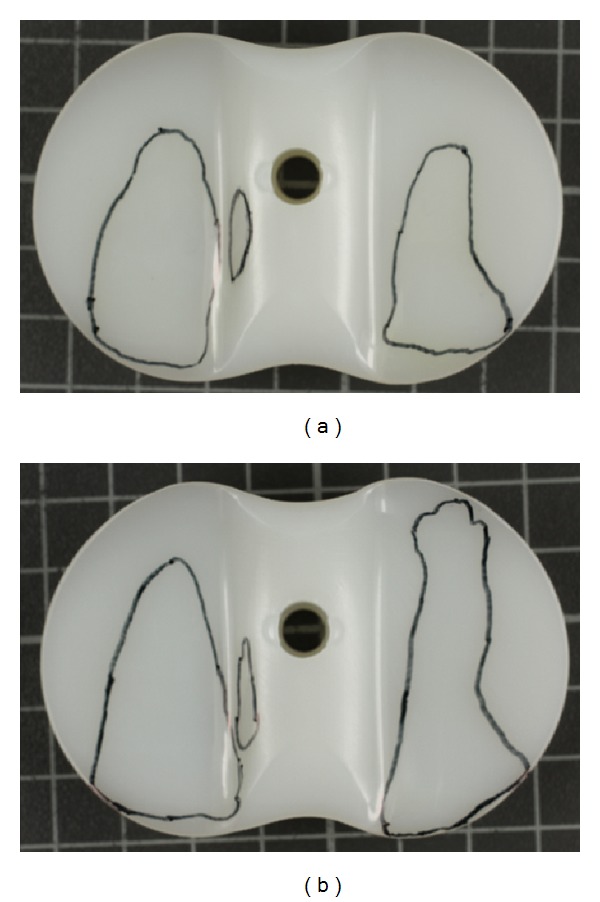
Wear areas on the PE for ligamentous-stable (a) and for ligamentous-unstable knee test conditions (b) (right knee in both figures).

Considerably higher kinematics was observed for the unstable knee compared to the stable knee (Figures 4 and 5). In comparison, internal/external rotation significantly increased from 12.62 ± 0.48° to 22.18 ± 4.48° (*P* ≤ 0.01). Anterior/posterior translation increased significantly from 9.46 ± 0.29 mm to 14.30 ± 2.03 mm (*P* ≤ 0.01). Higher rotations and translations for the unstable knee can be attributed to higher tibial internal rotation and higher tibial posterior translation during simulation. Wear areas are shown in Figure 6. Larger wear areas, particularly on the boundary areas (anterior and posterior) of the lateral plateau, were observed on the PE tested under ligamentously unstable knee conditions. No pitting or delamination was observed on the inserts.

**Figure 7:**
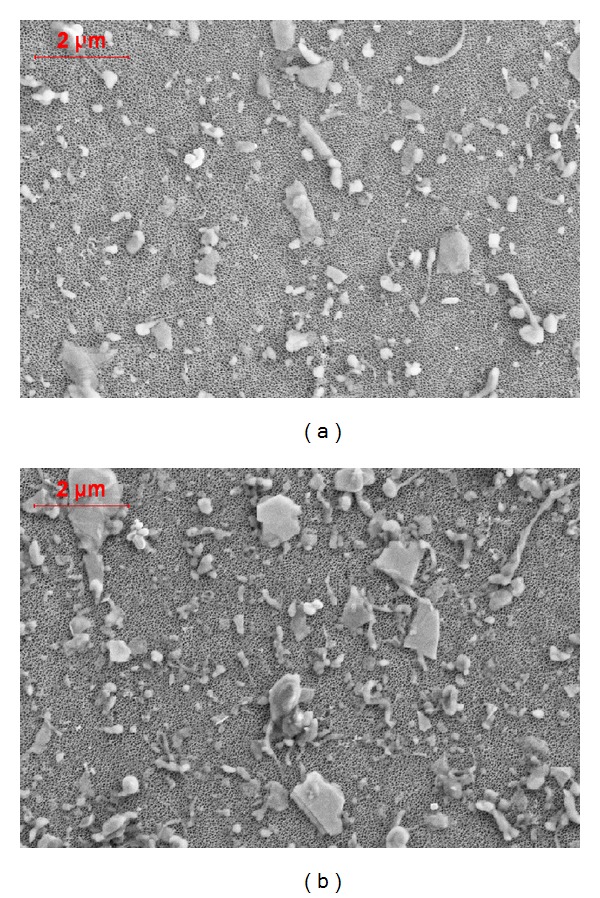
Example of analysed wear particles under ligamentous-stable (a) and for ligamentous-unstable knee test conditions (b). Relevant differences were observed in particular regarding the number of released wear particles.

Results of wear particle analysis are shown in Table 1. Wear particles are shown in Figure 7. Unstable knee conditions resulted in a higher number of generated wear particles (effect size 2.23). These particles were greater in size with a higher aspect ratio and a more irregular surface. However, only small effect sizes were determined for wear particle characteristics.

## 4. Discussion

In this study, the stabilization and wear behavior of a high-conforming knee design with two different ligament settings were simulated. Simulation of ligamentous-unstable TKR resulted in higher tibial posterior translation and higher tibial internal rotation.

It is known that anterior/posterior translations are mainly constrained by the cruciate ligaments [19, 28–30]. The ACL is the main constraint to tibial anterior translation, whereas the PCL is the main constraint to tibial posterior translation. The MCL is only minimally involved in translation restraint [29]. Higher tibial posterior translation is a consequence of the loss of PCL functionality simulated in unstable knee conditions. However, the high-conforming design was not capable of compensating for this loss of functionality of the PCL.

ACL and PCL participate only to a small extent in rotational stabilization of the knee joint, whereas MCL is a main stabilizer for rotational movements [29, 30]. Due to the insertion points and sense of rotation, the cruciate ligaments are not able to effectively counter rotational torques. In contrast, the collateral ligaments have an appropriate lever arm to withstand rotational torques [30]. Higher rotations under unstable conditions indicate that the high-conforming design was not capable of compensating for the loss of MCL functionality. The high-conforming design was more susceptible to insufficient rotational stabilization (76% increase) than to translation stabilization (51% increase) when comparing both test conditions.

Larger wear areas were observed on the lateral plateaus especially when testing unstable knee conditions. This may be related to the concept of wear simulation. Restraint during simulation is the sum of replicated passive structures, friction of the articulation, and restraint via implant design. Reducing the restraint of the passive structures during simulation will increase kinematics when no substituting via design or friction is occurring. Simulation is run with higher axial loading on the medial plateau. This results in smaller kinematics on the medial plateau (pivot point) and higher kinematics on the lateral plateau. This is a limitation of this study. During simulation only the restraint of the passive structures is replicated. However, ligamentously unstable conditions do alter not only restraint characteristics but also the mechanics (alignment and force transmission) of the joint, which has been neglected in this study.

Results showed that ligamentous-unstable TKR resulted in highly increased wear rates with an increased number of generated wear particles. The increased wear may be due to increased kinematics. Increased secondary movements, especially the cross-shear ratio [31–33], are known to be related to higher wear.

Retrieval analysis of high-conforming TKR has been associated with an increased risk of wear-related failure [34]. Higher delamination and pitting were observed for high-conforming inserts after a short mean implantation duration of 18.6 months. However, this analysis was based on gamma-in-air sterilized PE, which is known to be susceptible to high, oxidation-related wear. In our study, no delamination or pitting was observed after 5 million cycles under ligamentously adverse conditions. Five million cycles correspond to 1–3 years of* in vivo* use based on the activity of the patient [35, 36]. Therefore, this study may indicate an increase in PE quality due to improvements in manufacturing and sterilization techniques.

Under standard laboratory test conditions, it remains unclear whether conformity of knee designs results in an increased or decreased wear behavior [16–18]. Thus, the question arises if the increase in wear rates under unstable ligamentous conditions can be considered clinically critical. In a recent publication, Engh Jr. et al. [37] measured wear radiographically in failed and successful TKR. TKRs associated with a lower survivorship had a two-third higher wear rate. Taking this ratio into context of the increase in wear rate found in this study, the unstable TKR conditions may elicit a clinically critical wear performance. However, besides wear rates, biological reactions depend on wear particle characteristics like composition, morphology, and number of particles [38, 39]. Small differences were observed regarding wear characteristics (size and morphology), but relevant differences were particularly found in the total number of released particles. This increase (36%) was smaller than the increase in wear rates, relativizing the poor wear results under ligamentous-unstable test conditions.

Typically, wear testing of TKR is carried out according to ISO standards. In these ISO standards, only the cruciate ligaments (ACL/PCL) are considered (sacrificed/retained). This seems to be appropriate as the ACL is typically sacrificed during TKR implantation and the absence or insufficiency of the PCL is seen commonly in clinical settings. However, deficient ligamentous conditions are clinically often related to traumatic and degenerative changes. Changes to isolated structures, as defined by ISO, are rare. They would mostly occur in several structures (e.g., capsule, cruciate, and collateral ligaments) to varying extents [40–44]. Additionally, in TKR, soft tissues characteristics are altered due to chronic inflammation, chronic tibiofemoral malalignment, and ligament balancing during surgery [40]. Therefore, replication of the complex individual ligamentous interactions is difficult and complicates the establishment of a standardized yet clinically relevant wear test.

In this study, the unstable ligament model was chosen as the worst case scenario since (1) PCL is known for its restraining role in tibial posterior translation [19, 28–30] and (2) the MCL is known for its restraining role against tibial rotation [29]. Recently, the ligament restraint system of the previous ISO standard [45] has been modified. In the new ISO standard [46], a laxer and triphasenal (restraint in two motion directions and neutral zone) restraint model was defined, aiming to better replicate* in vivo* conditions. These more lax ligament characteristics are comparable to ligament characteristics defined in this study, despite both approaches (ISO and this study) replicating different ligament conditions. However, only limited published data is available for wear testing according to the newly introduced ISO standard [32, 47]. Haider et al. [47] reported a high wear rate of 19.88 mg/10^6^ cycles for a posterior-stabilized design without reporting the resulting kinematics. Recently, Grupp et al. [32] tested the wear behavior of a posterior-stabilized knee design, comparing the old ISO standard to the recently introduced one. A wear rate more than three times higher was reported when comparing the new to the old ISO standard. Additionally, significantly increased kinematics was observed. Kinematics increased for anterior/posterior translation by up to 41% and for internal/external rotation by up to 131%, when compared to the old, linear ISO standard. Depending on design features of the tested PS design, a mean wear rate of 17.1 mg/10^6^ cycles and 18.5 mg/10^6^ cycles was reported, which is comparable to the determined wear rate of the unstable knee in our study. However, when comparing kinematics to this study, the unstable knee resulted in kinematics approximately twice as high for translations as well as rotations. Thus, the resulting kinematics of this study is considered to be more critically than the observed increase in wear rates.

## 5. Conclusion

The tested high-conforming knee design resulted in increased tibial posterior translation and tibial internal rotations under ligamentous-unstable knee conditions. This can be related to insufficient stabilization via implant design. The tested design was not capable of compensating for the insufficient ligamentous stabilization.

The insufficient stabilization was accompanied by an increased wear related to higher kinematics. Increased wear rates and a higher number of wear particles of comparable size and morphology were observed under ligamentously unstable test conditions.

## Figures and Tables

**Table 1:** Results of wear particle analysis.

	Unstable knee	Stable knee	Effect size
Particles analysed	2016	1510	
Estimated number of particles per 10^6^ cycles	1.09 ∗ 10^12^ ± 0.14 ∗ 10^12^	0.80 ∗ 10^12^ ± 0.12 ∗ 10^12^	2.23
Equivalent circle diameter	0.263 ± 0.160 *µ*m	0.246 ± 0.162 *µ*m	0.11
Aspect ratio	1.776 ± 0.584	1.700 ± 0.504	0.14
Roundness	0.548 ± 0.151	0.577 ± 0.143	0.20
Form factor	0.657 ± 0.137	0.687 ± 0.120	0.23
